# Associations of Liver Function Parameters with New-Onset Hyperuricemia in a Large Taiwanese Population Study

**DOI:** 10.3390/nu14214672

**Published:** 2022-11-04

**Authors:** Chun-Chi Lu, Yi-Hsueh Liu, Wen-Hsien Lee, Szu-Chia Chen, Ho-Ming Su

**Affiliations:** 1 Department of Internal Medicine, Kaohsiung Municipal Siaogang Hospital, Kaohsiung Medical University, Kaohsiung 812, Taiwan; 2 Division of Cardiology, Department of Internal Medicine, Kaohsiung Medical University Hospital, Kaohsiung Medical University, Kaohsiung 807, Taiwan; 3 Faculty of Medicine, College of Medicine, Kaohsiung Medical University, Kaohsiung 807, Taiwan; 4 Division of Nephrology, Department of Internal Medicine, Kaohsiung Medical University Hospital, Kaohsiung Medical University, Kaohsiung 807, Taiwan; 5 Research Center for Precision Environmental Medicine, Kaohsiung Medical University, Kaohsiung 807, Taiwan

**Keywords:** new-onset hyperuricemia, total bilirubin, Taiwan Biobank

## Abstract

Hyperuricemia is the chief cause of gout and has been linked with hypertension, cardiovascular and renal disease, diabetes and metabolic syndrome. Liver with the highest protein expression of xanthine oxidase, the main enzyme responsible for uric acid formation, is the primary site of uric acid biosynthesis. However, there are few studies that examine the association between liver function and new-onset hyperuricemia. Hence, using the Taiwan Biobank dataset, we aimed to explore the capability of liver function parameters, including gamma-glutamyl transferase, total bilirubin, albumin, alanine aminotransferase and aspartate aminotransferase in association with the subsequent development of hyperuricemia. We analyzed 21,030 participants without hyperuricemia at baseline. Hyperuricemia was defined as a uric acid concentration > 6.0 mg/dL in women or >7.0 mg/dL in men. New-onset hyperuricemia was defined as participants without baseline hyperuricemia having developed hyperuricemia upon subsequent exam. Overall, 1804 (8.6%) of the study subjects developed new-onset hyperuricemia. After multivariable analysis, significant associations were found between the male sex (odds ratio [OR], 4.412; *p* < 0.001), high values of systolic blood pressure (SBP) (OR, 1.006; *p* = 0.012), body mass index (BMI) (OR, 1.064; *p* < 0.001), fasting glucose (OR, 1.005; *p* < 0.001), triglycerides (OR, 1.001; *p* = 0.003), uric acid (OR, 5.120; *p* < 0.001), low values of estimated glomerular filtration rates (eGFR) (OR, 0.995; *p* < 0.001), total bilirubin (OR, 0.616; *p* < 0.001) and new-onset hyperuricemia. The cutoff level of total bilirubin, according to the Youden index, of receiver operating characteristic curve for identifying new-onset hyperuricemia was 0.65 mg/dL. Low total bilirubin was defined as ≤0.65 mg/dL. After multivariable analysis, we found a significant association between low total bilirubin level (≤0.65 mg/dL) (OR = 0.806; *p* < 0.001) and new-onset hyperuricemia. Our present study demonstrated that in addition to male sex, high SBP, BMI, fasting glucose, triglycerides, and uric acid and low eGFR, the serum’s total bilirubin levels were negatively associated with new-onset hyperuricemia in a large Taiwanese cohort.

## 1. Introduction

Uric acid is the final product of purine catabolism, mostly produced in the liver and intestine. Chronic hyperuricemia might depend on the overproduction of uric acid and/or a reduced renal uric acid excretion [1]. Hyperuricemia is the chief cause of gout and has been linked with cardiovascular disease, hypertension, diabetes, renal disease, and metabolic syndrome [2,3,4,5]. Xanthine oxidase inhibitors are the safest and most effective uric acid-lowering drugs for the management of chronic hyperuricemia, while the efficacy of uricosuric agents is strongly modulated by pharmacogenetics [6]. Several parameters including increased body mass index (BMI), hyperglycemia, high blood pressure, and kidney disease contribute to hyperuricemia [7,8,9,10]. A previous study evaluated the short-term interaction between uric acid, low-density lipoprotein (LDL) cholesterol and incident hypertension, and found that the contemporary presence of suboptimal LDL-cholesterol and uric acid values is associated with an increased risk of developing hypertension [11]. Additionally, Cicero AFG, et al. investigated whether uric acid concentrations were positively associated with metabolic syndrome prevalence and middle-term (4-year) incidence in older overall healthy subjects, and found that hyperuricemia appears to be a highly prevalent component of MetS, especially in the most severe forms, as well as a risk factor for metabolic syndrome development [12].

The liver—with the highest protein expression of xanthine oxidase, the main enzyme responsible for uric acid formation—is the primary site of uric acid biosynthesis. In a longitudinal Chinese cohort study, Yang et al. evaluated the association between hyperuricemia and fatty liver disease and the temporal relationship between them, and found baseline hyperuricemia was associated with the new-onsetof fatty liver disease and baseline fatty liver disease was also associated with the subsequent development of hyperuricemia, a bidirectional relationship between fatty liver disease and hyperuricemia [13]. However, few studies have examined the association between liver function and new-onset hyperuricemia. Hence, using the Taiwan Biobank (TWB) dataset, we aimed to explore the capability of liver function parameters, including gamma-glutamyl transferase (GGT), total bilirubin, albumin, alanine aminotransferase (ALT) and aspartate aminotransferase (AST) to in association with the subsequent development of hyperuricemia.

## 2. Methods

### 2.1. Study Subjects

The TWB is the most extensive Taiwanese biobank, which collects data on lifestyle factors and genomics of healthy volunteers aged 30–70 years [14,15]. All of study subjects underwent physical examinations and provided blood samples. The participants who enroll in the TWB follow up after 2–4 years. Information, including a questionnaire, physical examination and blood examination, is collected upon first enrollment. The study subjects needed to answer questionnaires, and a TWB researcher provided assistance when required. Personal information and medical histories were also recorded.

TWB enrolled a total of 104,451 participants from 2012 to 2018. Personal information was recorded including age, sex, resting heart rate, systolic/diastolic blood pressure (SBP/DBP), BMI, and presence of diabetes mellitus (DM) and hypertension. Overnight fasting blood data were also recorded, such as fasting glucose, total cholesterol, triglycerides, hemoglobin, serum creatinine, GGT, total bilirubin, albumin, ALT, and AST. We used four-variable modification of diet in renal disease formula to calculate estimated glomerular filtration rate (eGFR) [16].

### 2.2. Definition of New-Onset Hyperuricemia

A uric acid concentration of >6.0 mg/dL in women or >7.0 mg/dL in men was taken to indicate hyperuricemia [17]. New-onset hyperuricemia was defined as participants without baseline hyperuricemia developing hyperuricemia during the subsequent exam.

### 2.3. Ethics Statement

All of our study subjects provided written informed consent. The Institutional Review Board (IRB) of Kaohsiung Medical University Hospital approved our study design (KMUHIRB-E(I)-20210058). The ethical approval of TWB was granted by the IRB on Biomedical Science Research, Academia Sinica, Taiwan and the Ethics and Governance Council of the TWB. Additionally, the study was conducted according to the Declaration of Helsinki.

### 2.4. Statistical Analysis

Statistical analyses were performed using SPSS version 22.0 (IBM Inc., Armonk, NY, USA). Categorical variables are shown as percentages, and continuous variables are shown as mean ± standard deviation. Differences in categorical variables were analyzed using the chi-square test, while differences in continuous variables were analyzed using independent samples *t* test. The normal distribution of variables was evaluated with the Kolmogorov–Smirnov test. Homogeneity of variance was tested with Levene’s test. Levene’s test was used to assess the equality of variances, and an independent sample *t*-test. A two-tailed *p* value less than 0.05 was considered statistically significant. Binary logistic regression analysis was performed to examine the associations between the studied parameters and new-onset hyperuricemia. Results were considered significant at *p* < 0.05.

## 3. Results

**Figure 1:**
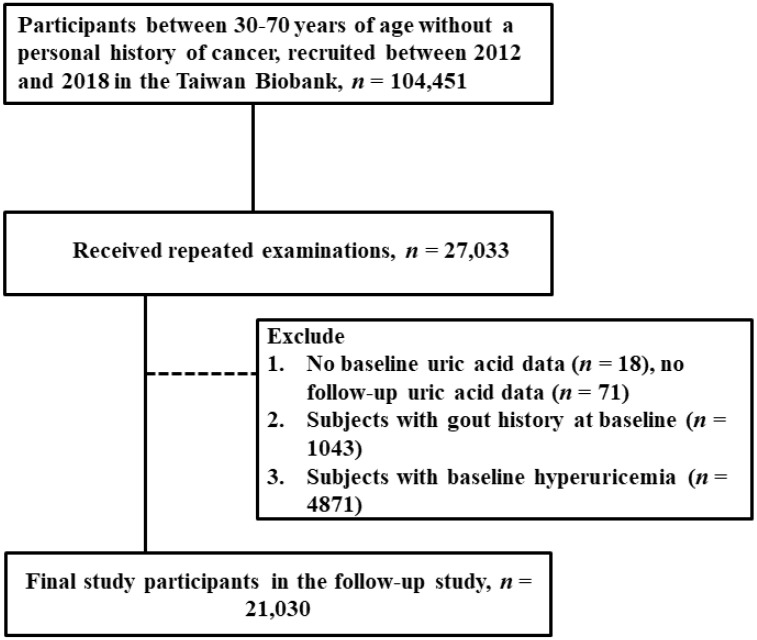
Flowchart of study population.

This study aimed to identify determinants of future hyperuricemia development; we only selected study subjects with complete baseline and subsequent exam data (*n* = 27,033). We excluded participants without baseline uric acid data (*n* = 18), participants without subsequent exam uric acid data (*n* = 71), participants with gout history at baseline (*n* = 1043), and participants with baseline hyperuricemia (*n* = 4871). Finally, a total of 21,030 participants formed our study population (Figure 1). Of a total 21,030 study subjects, there were 6286 male (30%) and 14,744 (70%) female participants, respectively.

During the subsequent exam, 1804 of the study subjects, (8.6%) developed new-onset hyperuricemia from the total of 21,030 study subjects. Of the 6286 male participants, 716 subjects (11.4%) developed new-onset hyperuricemia. Of 14,744 female participants, 1088 subjects (7.4%) developed new-onset hyperuricemia. Table 1 shows comparisons of baseline variables between participants with (*n* = 1804) and without (*n* = 19,226) new-onset hyperuricemia. The participants with new-onset hyperuricemia were older, had increased rates of hypertension and DM, elevated SBP, DBP, BMI, fasting glucose, total cholesterol, hemoglobin, triglyceride, uric acid, GGT, albumin, ALT and AST, and a lower eGFR and total bilirubin than those without new-onset hyperuricemia.

Table 2 shows the determinants of new-onset hyperuricemia in the 21,030 participants in binary logistic analysis. Univariable analysis showed associations between old age, male sex, DM, hypertension, high SBP, high DBP, high BMI, high fasting glucose, high total cholesterol, high triglycerides, high hemoglobin, high uric acid, low eGFR, high GGT, low total bilirubin, high albumin, high ALT and high AST and new-onset hyperuricemia. After multivariable analysis, male sex, high values of SBP, BMI, fasting glucose, triglyceride and uric acid; and low values of eGFR and total bilirubin were significantly associated with new-onset hyperuricemia.

The cutoff level of total bilirubin according to the Youden index of receiver operating characteristic curve to identify new-onset hyperuricemia is 0.65 mg/dL. Low total bilirubin was defined as ≤0.65 mg/dL. We found a significant association between low total bilirubin level (≤0.65 mg/dL) (odds ratio = 0.806; 95% confidence interval = 0.722–0.901; *p* < 0.001) and new-onset hyperuricemia.

The determinants of new-onset hyperuricemia in binary logistic analysis in the 20,303 participants with normal total bilirubin (≤1.2 mg/dL) are shown in Table 3. In univariable analysis, old age, male sex, DM, hypertension, elevated values of SBP, DBP, BMI, fasting glucose, total cholesterol, triglycerides, hemoglobin and uric acid, decreased eGFR, high values of GGT, albumin ALT and AST, and low total bilirubin were correlated with new-onset hyperuricemia. Multivariable analysis showed significant associations between male sex, high values of SBP, fasting glucose, BMI, triglyceride and uric acid, low eGFR, and low total bilirubin with new-onset hyperuricemia. The determinants of new-onset hyperuricemia in subgroup participants with normal total bilirubin were the same as those in total participants.

## 4. Discussion

In this study, we found that in addition to male sex, high values of SBP, BMI, fasting glucose, triglyceride, and uric acid and low value of eGFR, low serum total bilirubin were significantly associated with new-onset hyperuricemia both in total participants and in participants with normal total bilirubin after multivariable adjustment.

Hypertension has been closely associated with hyperuricemia [18]. In the present study, high SBP was associated with new-onset hyperuricemia, so patients with elevated SBP might have an increased future risk of developing hyperuricemia.

Li et al. included 10,611 hypertensive patients and evaluated the risk factors of new-onset hyperuricemia and found increased BMI was significantly associated with an increased risk of new-onset hyperuricemia [19]. Although there were only 10.3% subjects with hypertension in our study, our result still demonstrated high BMI was associated with future development of hyperuricemia.

Yang et al. evaluated the prevalence of hyperuricemia and its relationship with serum lipids and blood glucose in a cross-sectional study and found hyperuricemia was closely linked to serum lipids and blood fasting glucose [20]. Our present study also showed high fasting glucose and triglyceride at baseline were associated with new-onset hyperuricemia after subsequent exam. Hence, fasting glucose and triglyceride were helpful parameters in association with future hyperuricemia development.

Oliveira et al. reported an independent inverse association between uric acid and eGFR in young Brazilian adults [21]. Zhou et al. reported that increasing uric acid was an independent risk factor for kidney disease and rapid decline in eGFR, and that decreased uric acid could reduce these risks in Chinese subjects [22]. In the present study, we also found a negative correlation between eGFR and uric acid at baseline after multivariable analysis. Furthermore, we demonstrated low eGFR was associated with new-onset hyperuricemia after subsequent exam.

Our study shows that total bilirubin is negatively associated with new-onset hyperuricemia. A previous cross-sectional study showed that comparing subjects with morbid obesity without hyperuricemia, subjects with morbid obesity and hyperuricemia had an increase of total bilirubin value [23]. Our result demonstrated that low baseline total bilirubin level was associated with new-onset hyperuricemia after adjusting many important confounding factors. Hyperuricemia, a metabolic disease, was closely correlated to oxidative stress and inflammatory responses and caused by reduced excretion or increased production of uric acid [24]. Bilirubin was an end product of heme degradation and previous studied demonstrated that at high concentrations, bilirubin was cytotoxic, which could lead to brain damage, but at low concentrations, it could serve as an endogenous antioxidant [25,26]. In our present study, we found a significant association between low total bilirubin level (≤0.65 mg/dL) and new-onset hyperuricemia both in total population and those with normal total bilirubin level. The exact mechanism was not clear, but the anti-oxidant effect of bilirubin at low concentration might partially explain this result.

## 5. Limitations

Several limitations to this study should be noted. First, the TWB does not include information on urate- or lipid-lowering drugs, anti-diabetic drugs, or anti-hypertensive drugs, all of which could have affected the prevention or development of new-onset hyperuricemia, blood pressure, lipid profiles, and fasting glucose, and consequently the association between factors and new-onset hyperuricemia could be under-estimated. Second, our study subjects only received survey and examinations at baseline and after subsequent exam, only two examinations, so the exact time of hyperuricemia onset was unknown. Third, we had no data of physical activity and dietary habits, which might have affected the serum uric acid value. Fourth, the participants in this study were of Chinese ethnicity, and thus our findings may not be generalizable to other ethnicities. Fifth, several genetic polymorphisms of xanthine oxidoreductase had been identified [4,5], but we had no genetic data to assess the relationship between new-onset hyperuricemia and genetic variations. In addition, we had no data of direct and indirect bilirubin values, so we could not evaluate the association of these two parameters with new-onset hyperuricemia. Finally, according to the statistics from the TWB, about 25% of the participants return for subsequent exam assessments, which may have resulted in sample bias.

## 6. Conclusions

Our present study demonstrated that in addition to male sex, high values of uric acid, SBP, BMI, fasting glucose, and triglyceride and low value of eGFR, serum total bilirubin levels were negatively associated with new-onset hyperuricemia in a large Taiwanese cohort.

## Figures and Tables

**Table 1:** Comparison of baseline characteristics between participants with and without new-onset hyperuricemia.

Characteristics	Participants with New-Onset Hyperuricemia (*n* = 1804)	Participants without New-Onset Hyperuricemia (*n* = 19,226)	*p* Value	All Participants (*n* = 21,030)
Age (year)	52 ± 10	51 ± 10	<0.001	51 ± 10
Male sex (%)	40	29	<0.001	30
Diabetes mellitus (%)	7.3	4.4	<0.001	4.7
Hypertension (%)	17.5	9.6	<0.001	10.3
Systolic blood pressure (mmHg)	122 ± 18	115 ± 17	<0.001	116 ± 17
Diastolic blood pressure (mmHg)	75 ± 11	71 ± 11	<0.001	71 ± 11
Heart rate (beat/min)	69.2 ± 9.6	69.4 ± 8.9	0.201	69.4 ± 9.0
Body mass index (kg/m^2^)	25.1 ± 3.4	23.3 ± 3.2	<0.001	23.5 ± 3.3
Fasting glucose (g/dL)	99 ± 25	95 ± 20	<0.001	95 ± 20
Total cholesterol (mg/dL)	197 ± 35	194 ± 35	0.001	194 ± 35
Triglyceride (mg/dL)	129 ± 81	101 ± 72	<0.001	104 ± 73
Hemoglobin (g/dL)	13.9 ± 1.5	13.5 ± 1.5	<0.001	13.6 ± 1.5
eGFR (mL/min/1.73 m^2^)	105 ± 24	114 ± 25	<0.001	113 ± 25
Uric acid (mg/dL)	5.8 ± 0.8	4.9 ± 1.0	<0.001	5.0 ± 1.0
Liver-related parameters				
AST (μ/L)	25.0 ± 12.2	23.8 ± 11.0	<0.001	23.9 ± 11.1
ALT (μ/L)	24.7 ± 17.50	21.6 ± 17.6	<0.001	21.9 ± 17.6
Albumin (g/dL)	4.56 ± 0.23	4.54 ± 0.23	<0.001	4.54 ± 0.23
Total bilirubin (mg/dL)	0.64 ± 0.28	0.66 ± 0.27	0.001	0.66 ± 0.27
GGT (μ/L)	26 ± 26	21 ± 24	<0.001	21 ± 24

eGFR, estimated glomerular filtration rate; AST, aspartate aminotransferase; ALT, alanine aminotransferase; GGT, gamma-glutamyl transpeptidase.

**Table 2:** Determinants of new-onset hyperuricemia using binary logistic analysis in all 21,030 study participants.

Parameter	Univariable		Multivariable	
	OR (95% CI)	*p*	OR (95% CI)	*p*
Age (per 1 year)	1.02 (1.01–1.02)	<0.001	1.00 (0.99–1.01)	0.66
Male (vs. female)	1.61 (1.46–1.78)	<0.001	4.41 (3.71–5.24)	<0.001
Diabetes mellitus	1.70 (1.41–2.06)	<0.001	1.11 (0.87–1.42)	0.39
Hypertension	1.99 (1.75–2.27)	<0.001	1.13 (0.97–1.32)	0.12
Systolic blood pressure (per 1 mmHg)	1.02 (1.02–1.02)	<0.001	1.01 (1.00–1.01)	0.01
Diastolic blood pressure (per 1 mmHg)	1.03 (1.03–1.04)	<0.001	1.00 (1.00–1.01)	0.50
Heart rate (per 1 beat/min)	1.00 (0.99–1.00)	0.20		
Body mass index (per 1 kg/m^2^)	1.16 (1.14–1.17)	<0.001	1.06 (1.05–1.08)	<0.001
Fasting glucose (per 1 g/dL)	1.01 (1.01–1.01)	<0.001	1.01 (1.00–1.01)	<0.001
Total cholesterol (per 1 mg/dL)	1.00 (1.00–1.00)	0.001	1.00 (1.00–1.00)	0.06
Triglyceride (per 1 mg/dL)	1.00 (1.00–1.00)	<0.001	1.00 (1.00–1.00)	0.01
Hemoglobin (per 1 g/dL)	1.18 (1.15–1.22)	<0.001	0.98 (0.94–1.03)	0.49
Uric acid (per 1 mg/dL)	3.04 (2.86–3.23)	<0.001	5.12 (4.64–5.65)	<0.001
eGFR (per 1 mL/min/1.73 m^2^)	0.98 (0.98–0.99)	<0.001	1.00 (0.99–1.00)	<0.001
Liver-related parameters				
AST (per 1 μ/L)	1.01 (1.00–1.01)	<0.001	1.01 (1.00–1.01)	0.27
ALT (per 1 μ/L)	1.01 (1.01–1.01)	<0.001	0.99 (0.99–1.00)	0.06
Albumin (per 1 g/dL)	1.53 (1.23–1.90)	<0.001	1.08 (0.84–1.39)	0.55
Total bilirubin (per 1 mg/dL)	0.73 (0.60–0.88)	0.01	0.62 (0.50–0.76)	<0.001
GGT (per 1 μ/L)	1.01 (1.00–1.01)	<0.001	1.00 (1.0–1.00)	0.50

Values expressed as odds ratio (OR) and 95% confidence interval (CI). Abbreviations are the same as in Table 1.

**Table 3:** Determinants of new-onset hyperuricemia using binary logistic analysis in 20,303 study participants with normal total bilirubin (≤1.2 mg/dL).

Parameter	Univariable		Multivariable	
	OR (95% CI)	*p*	OR (95% CI)	*p*
Age (per 1 year)	1.02 (1.01–1.02)	<0.001	1.00 (1.00–1.01)	0.48
Male (vs. female)	1.64 (1.48–1.81)	<0.001	4.27 (3.59–5.08)	<0.001
Diabetes mellitus	1.73 (1.42–2.09)	<0.001	1.13 (0.88–1.44)	0.34
Hypertension	2.03 (1.77–2.31)	<0.001	1.15 (0.99–1.35)	0.07
Systolic blood pressure (per 1 mmHg)	1.02 (1.02–1.02)	<0.001	1.01 (1.00–1.01)	0.03
Diastolic blood pressure (per 1 mmHg)	1.03 (1.03–1.04)	<0.001	1.00 (1.00–1.01)	0.27
Heart rate (per 1 beat/min)	1.00 (0.99–1.00)	0.255		
Body mass index (per 1 kg/m^2^)	1.16 (1.14–1.17)	<0.001	1.06 (1.05–1.08)	<0.001
Fasting glucose (per 1 g/dL)	1.01 (1.01–1.01)	<0.001	1.01 (1.00–1.01)	0.01
Total cholesterol (per 1 mg/dL)	1.00 (1.00–1.00)	0.001	1.00 (1.00–1.00)	0.09
Triglyceride (per 1 mg/dL)	1.00 (1.00–1.00)	<0.001	1.00 (1.00–1.00)	0.01
Hemoglobin (per 1 g/dL)	1.19 (1.15–1.23)	<0.001	0.98 (0.94–1.03)	0.50
Uric acid (per 1 mg/dL)	3.06 (2.87–3.25)	<0.001	5.02 (4.55–5.54)	<0.001
eGFR (per 1 mL/min/1.73 m^2^)	0.98 (0.98–0.99)	<0.001	1.00 (0.99–1.00)	<0.001
Liver-related parameters				
AST (per 1 μ/L)	1.01 (1.01–1.01)	<0.001	1.00 (0.99–1.01)	0.53
ALT (per 1 μ/L)	1.01 (1.01–1.01)	<0.001	1.00 (0.99–1.00)	0.15
Albumin (per 1 g/dL)	1.54 (1.24–1.92)	<0.001	1.10 (0.85–1.42)	0.48
Total bilirubin (per 1 mg/dL)	0.63 (0.50–0.81)	<0.001	0.51 (0.39–0.67)	<0.001
GGT (per 1 μ/L)	1.01 (1.01–1.01)	<0.001	1.00 (1.00–1.00)	0.41

Values expressed as odds ratio (OR) and 95% confidence interval (CI). Abbreviations are the same as in Table 1.

## Data Availability

The data underlying this study are from the Taiwan Biobank. Due to restrictions placed on the data by the Personal Information Protection Act of Taiwan, the minimal data set cannot be made publicly available. Data may be available upon request to interested researchers. Please send data requests to: Szu-Chia Chen. Division of Nephrology, Department of Internal Medicine, Kaohsiung Medical University Hospital, Kaohsiung Medical University.
